# Supplementary material for: Hypoxia-induced metastatic heterogeneity in pancreatic cancer
Source: Res Sq. 2025 Oct 1:rs.3.rs-7394295. Preprint. [Version 1] doi: 10.21203/rs.3.rs-7394295/v1 (PMC12622147; doi:10.21203/rs.3.rs-7394295/v1)
Supplement: Supplement 1 [file NIHPPrs7394295v1-supplement-1.pdf]

Figure S1

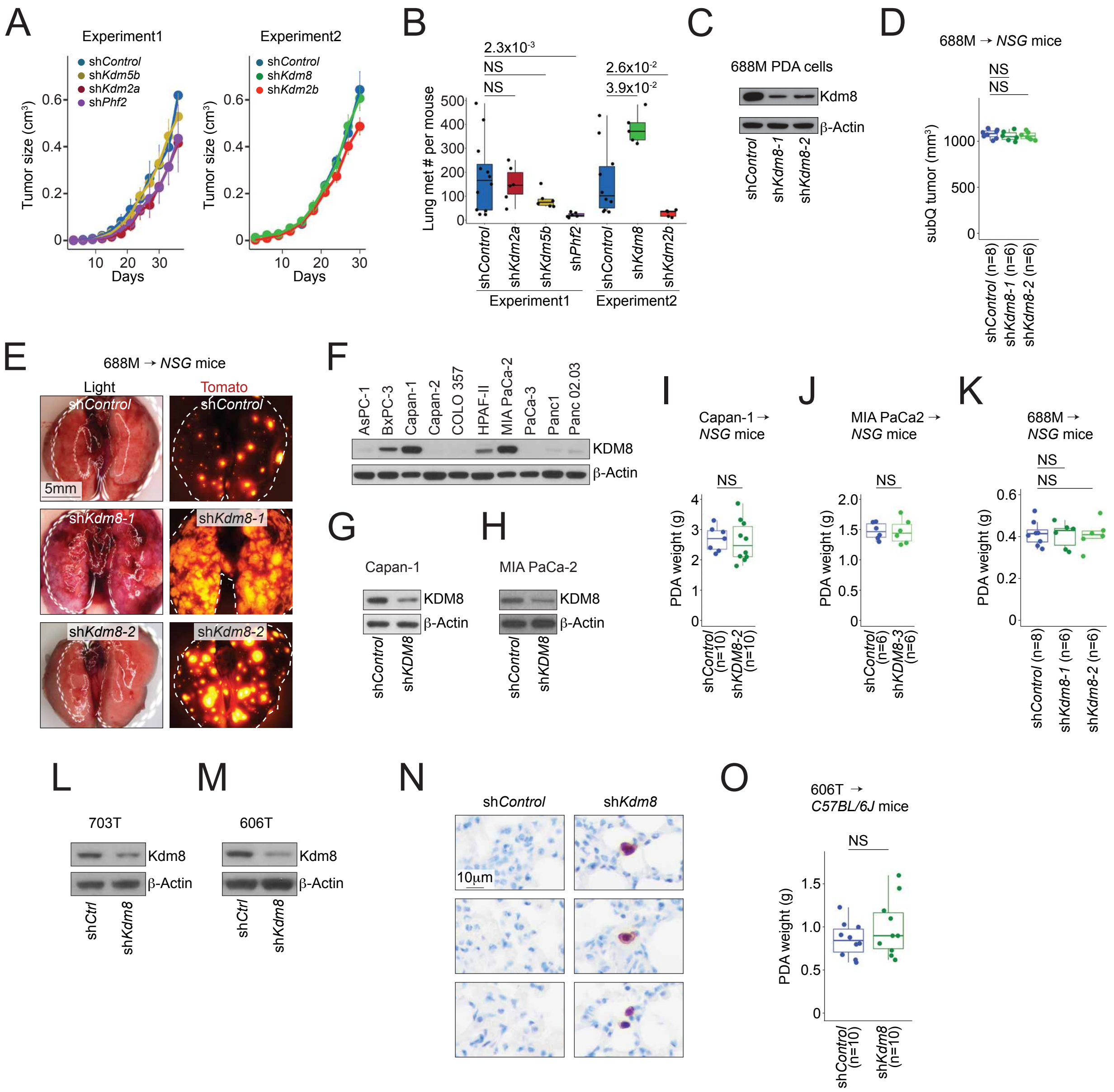

Figure S1, related to Figure 1.

(A,B) Subcutaneous tumor growth (A) and counts of lung metastases (B) seeded from the subcutaneous tumors in the *NSG* mice receiving the Tomato-positive 688M PDA cells transduced with control (sh*Control*, experiment 1, n=12; experiment 2, n=10) or shRNA targeting the indicated *Kdm* genes (sh*Kdm2a*, n=6; sh*Kdm5b*, n=6; sh*Phf2*, n=6; sh*Kdm8*, n=5; sh*Kdm2b*, n=5). Two independent experiments are shown. (C) *Kdm8* knockdown in 688M cells using two independent shRNAs. (D,E) Subcutaneous tumor size (D) and representative light (left) and fluorescent (right) images of the lungs (E) upon sacrifice for the tumor studies in Figure S1B. (F) Immunoblot for the endogenous abundance of KDM8 and β-Actin in the indicated human PDA cell lines. (G,H) *KDM8* knockdown in Capan-1 (G) and MIA PaCa-2 (H) human PDA cells using a *KDM8*-targeting shRNA. (I-K) The primary PDA tumor weight for the orthotopic tumor studies using Capan-1, MIA PaCa-2, and 688M PDA cells. (L,M) *Kdm8* knockdown in 703T (L) and 606T (M) murine PDA cells using a *Kdm8*-targeting shRNA. (N) Representative Tomato IHC images demonstrating individual micrometastatic PDA cells in the lungs of the *B6* mice receiving sh*Kdm8*-expressing 606T PDA cells (right) compared to control (left). (O) The primary PDA tumor weight in each *B6* mouse orthotopically transplanted with 606T PDA cells as in Figures 1N-1P. NS, not significant. Each dot is a mouse and boxes represent medians with interquartile range between 25th and 75th percentiles.

Figure S2

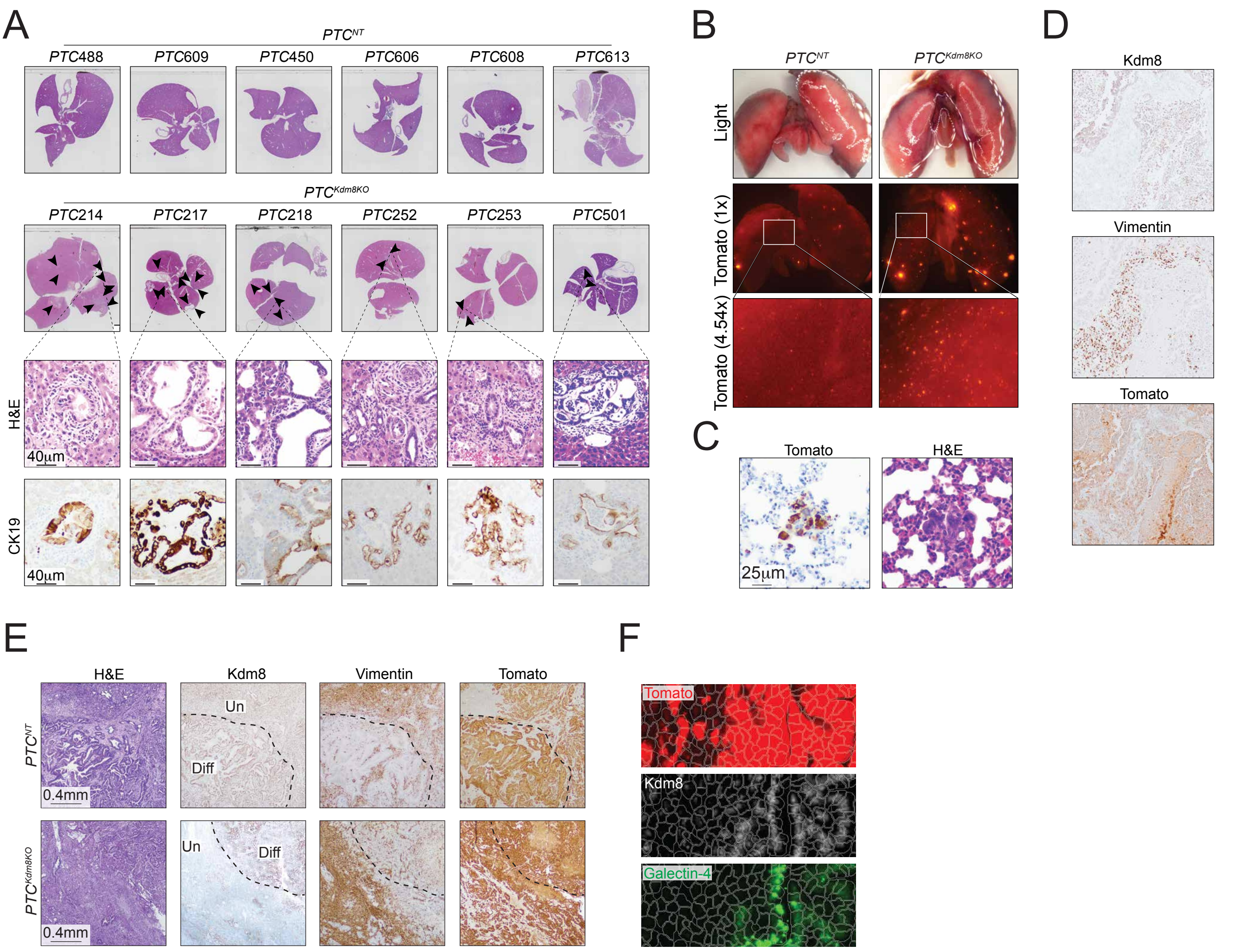

Figure S2, related to Figure 2.
(A) Haematoxylin and eosin staining (H&E) and CK19 immunohistochemistry (IHC) of liver samples from *PTC*^{NT} (top row) and *PTC*^{Kdm8KO} (2nd, 3rd, and 4th rows) mice. (B) Representative light (top) and fluorescent images of the whole lungs (2nd row) and zoom-in regions (3rd row) from a *PTC*^{NT} (left) and a *PTC*^{Kdm8KO} (right) mouse. (C) Representative H&E (right) and Tomato IHC (left) of lung micrometastases in a *PTC*^{Kdm8KO} mouse. (D) Representative IHC of Kdm8, Vimentin, and Tomato of serial sections from a primary PDA region as in Figure 2M. (E) Representative H&E and IHC of Kdm8, Vimentin, and Tomato of serial sections from primary PDA tumors of a *PTC*^{NT} (top) and a *PTC*^{Kdm8KO} (bottom) mouse. Dotted lines demarcate the fully differentiated (diff) and poorly differentiated (Un) regions. (F) Representative images as in Figure 2I of a *PTC*^{Kdm8KO} tumor with automated cell segmentation, overlaid with pseudocolors for Tomato, Kdm8, and Galectin-4 (top to bottom).

Figure S3

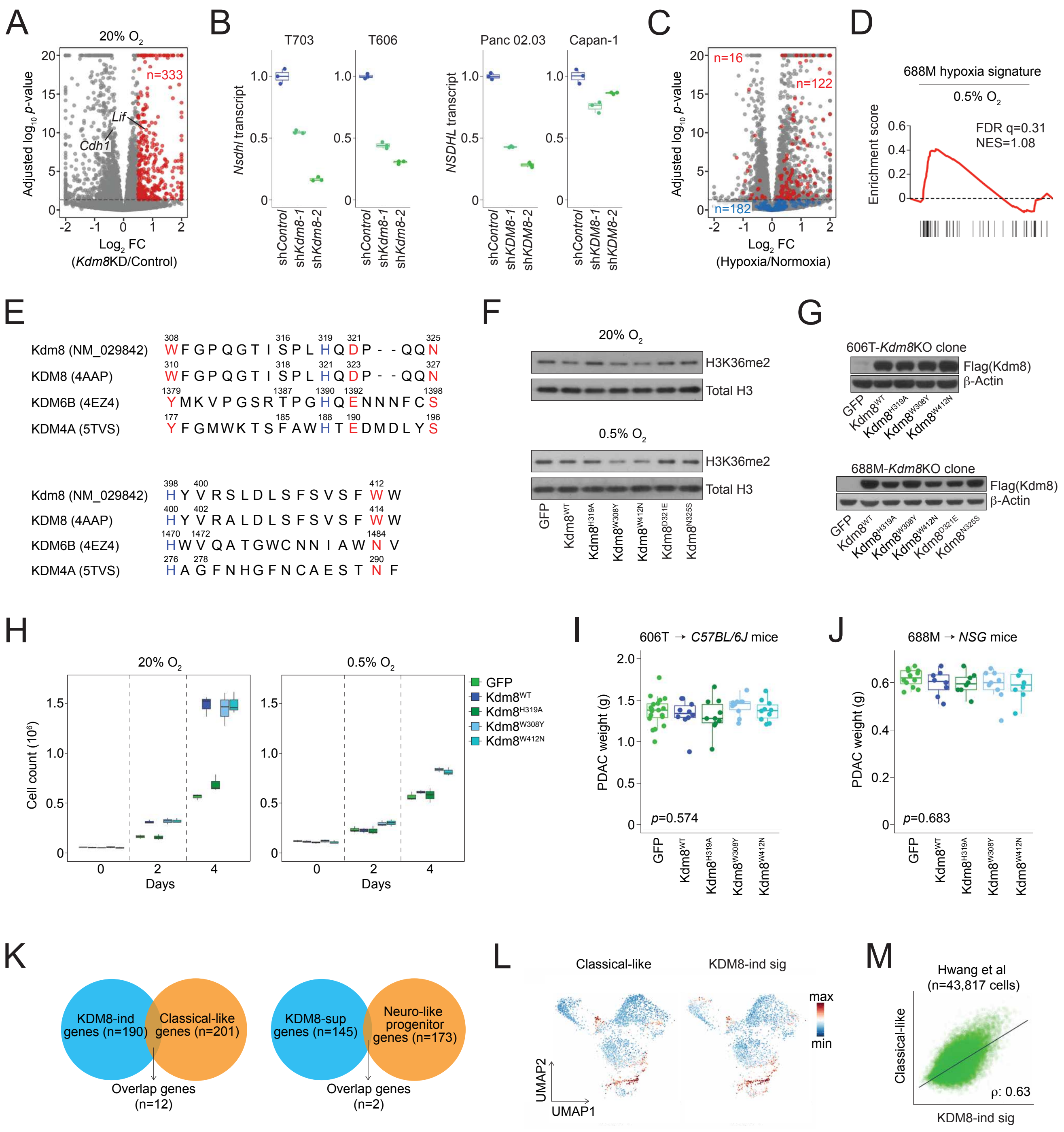
A
20% O2
n=333
Lif
Cdh1
Adjusted log10 p-value
Log2 FC (Kdm8KD/Control)
B
T703
T606
Panc 02.03
Capan-1
Nsdhl transcript
NSDHL transcript
shControl
shKdm8-1
shKdm8-2
shKDM8-1
shKDM8-2
C
n=16
n=122
n=182
Adjusted log10 p-value
Log2 FC (Hypoxia/Normoxia)
D
688M hypoxia signature
0.5% O2
FDR q=0.31
NES=1.08
Enrichment score
E
Kdm8 (NM_029842) WFGPQGTISPLHQDP--QQN
KDM8 (4AAP) WFGPQGTISPLHQDP--QQN
KDM6B (4EZ4) YMKVPGSRTPGHQENNNFCS
KDM4A (5TVS) YFGMWKTSFAWHTEDMDLYS
Kdm8 (NM_029842) HYVRSLDLSFSVSFWW
KDM8 (4AAP) HYVRALDLSFSVSFWW
KDM6B (4EZ4) HWVQATGWCNNIAWNV
KDM4A (5TVS) HAGFNHGFNCAESTNF
F
20% O2
0.5% O2
H3K36me2
Total H3
GFP
Kdm8WT
Kdm8H319A
Kdm8W308Y
Kdm8W412N
Kdm8D321E
Kdm8N325S
G
606T-Kdm8KO clone
688M-Kdm8KO clone
Flag(Kdm8)
β-Actin
H
20% O2
0.5% O2
Cell count (10^6)
Days
GFP
Kdm8WT
Kdm8H319A
Kdm8W308Y
Kdm8W412N
I
606T → C57BL/6J mice
PDAC weight (g)
p=0.574
J
688M → NSG mice
PDAC weight (g)
p=0.683
K
KDM8-ind genes (n=190)
Classical-like genes (n=201)
Overlap genes (n=12)
KDM8-sup genes (n=145)
Neuro-like progenitor genes (n=173)
Overlap genes (n=2)
L
Classical-like
KDM8-ind sig
max
min
UMAP2
UMAP1
M
Hwang et al (n=43,817 cells)
Classical-like
KDM8-ind sig
ρ: 0.63

Figure S3, related to Figure 3.

(A) Volcano plot of differential genes induced upon *Kdm8* knockdown in normoxia (n=333 genes upregulated, red). *Lif* and *Cdh1* are highlighted for Figure 5F. (B) Expression of *Nsdhl* and *NSDHL* following *Kdm8* and *KDM8* knockdown the indicated PDA cell lines, as measured by quantitative PCR. (C) Volcano plot of differential genes induced or suppressed by hypoxia in 688M cells. Genes that are induced by *Kdm8* knockdown are highlighted (red, adjusted p-value < 0.05; blue, non-significant under hypoxia). (D) GSEA of the 688M hypoxia gene signature enriched in *Kdm8* knockdown 688M cells cultured in hypoxia. (E) Alignment of the JmjC domains of human KDM8, KDM6B, and KDM4A (with PDB identifiers). Red, non-conserved residues that share consensus in KDM6B and KDM4A; blue, conserved residues among all three KDMs. Murine Kdm8 is shown for comparison. KDM8 residue 328-399 are omitted due to the absence of conserved residues in KDM6B and KDM4A, and their high divergence within KDM8. (F) Immunoblots of H3K36me2 and total H3K36 in *Kdm8* knockout 688M cells re-expressing the indicated Kdm8 variants cultured in normoxia (top) or hypoxia (bottom). (G) Immunoblots of indicated Flag tagged Kdm8 variants in *Kdm8*-knockout 606T (top) and 688M (bottom) clones. β-actin shows equal loading. (H) Cell counts of *Kdm8* knockout 688M cells re-expressing the indicated Kdm8 variants cultured in normoxia (left) or hypoxia (right) following 0, 2, and 4 days post seeding. Comparable results were observed using 606T cells (not shown). (I,J) Pancreatic tumor weights at the time of sacrifice for the tumor studies shown in Figures 3E and 3F, respectively. (K) Venn diagrams demonstrating the shared genes between the KDM8-indued and the classical gene signatures and those between the KDM8-suppressed and the neuro-like progenitor (Hwang et al, 2022 Nat Genet) gene signatures. (L,M) UMAP demonstrating human PDA malignant cells defined in Hwang et al, 2022 Nat Genet colored by the KDM8-induced and classical gene signatures (L). The relationship between the scores of the two gene signatures is shown with the Pearson correlation coefficient ρ (M).

Figure S4

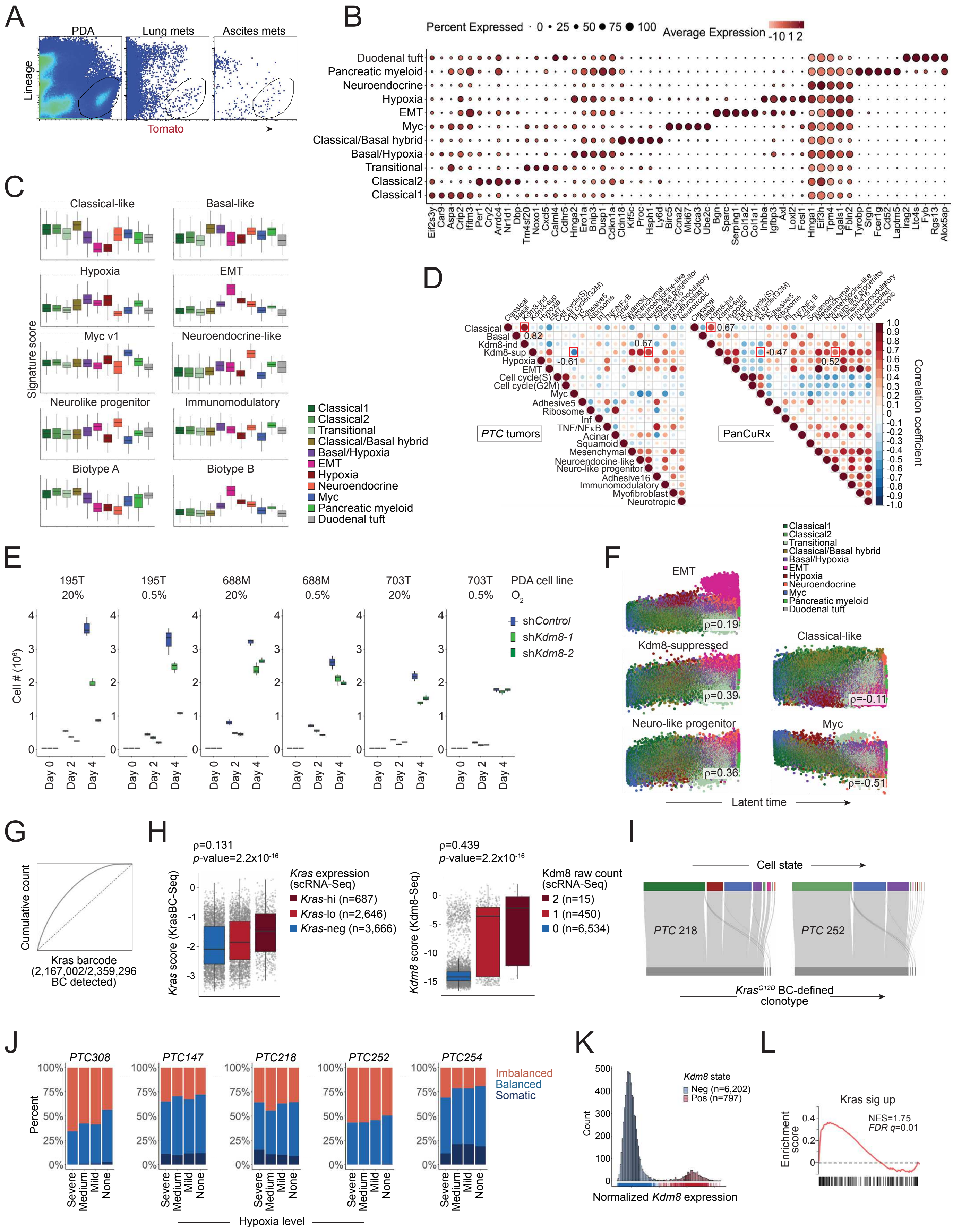
A
PDA
Lung mets
Ascites mets
Lineage
Tomato
B
Percent Expressed · 0 • 25 • 50 • 75 • 100
Average Expression -1 0 1 2
Duodenal tuft
Pancreatic myeloid
Neuroendocrine
Hypoxia
EMT
Myc
Classical/Basal hybrid
Basal/Hypoxia
Transitional
Classical2
Classical1
Eif2s3y Car9 Aspa Crip2 Ifitm3 Per1 Cry2 Arrdc4 Nr1d1 Dbp Tm4sf20 Noxo1 Cxcl5 Calml4 Cdhr5 Hmga2 Ero1a Bnip3 Dusp1 Cdkn1a Cldn18 Klf5c Proc Hsph1 Ly6d Birc5 Ccna2 Mki67 Cdca3 Ube2c Bgn Sparc Serping1 Col1a2 Col1a1 Inhba Igfbp3 Axl Loxl2 Fosl1 Hmga1 Eif3h Tpm4 Lgals1 Fbln2 Tyrobp Srgn Fcer1g Cd52 Laptm5 Irag2 Ltc4s Fyb Rgs13 Alox5ap
C
Classical-like
Basal-like
Hypoxia
EMT
Myc v1
Neuroendocrine-like
Neurolike progenitor
Immunomodulatory
Biotype A
Biotype B
Signature score
Classical1
Classical2
Transitional
Classical/Basal hybrid
Basal/Hypoxia
EMT
Hypoxia
Neuroendocrine
Myc
Pancreatic myeloid
Duodenal tuft
D
Classical
Basal
Kdm8-ind
Kdm8-sup
Hypoxia
EMT
Cell cycle(S)
Cell cycle(G2M)
Myc
Adhesive5
Ribosome
Inf
TNF/NFκB
Acinar
Squamoid
Mesenchymal
Neuroendocine-like
Neuro-like progenitor
Adhesive16
Immunomodulatory
Myofibroblast
Neurotropic
0.82
0.67
-0.61
0.67
-0.47
0.52
PTC tumors
PanCuRx
Correlation coefficient
1.0 0.9 0.8 0.7 0.6 0.5 0.4 0.3 0.2 0.1 0 -0.1 -0.2 -0.3 -0.4 -0.5 -0.6 -0.7 -0.8 -0.9 -1.0
E
195T 20%
195T 0.5%
688M 20%
688M 0.5%
703T 20%
703T 0.5%
PDA cell line
O_2
shControl
shKdm8-1
shKdm8-2
Cell # (10^6)
Day 0
Day 2
Day 4
F
EMT
ρ=0.19
Kdm8-suppressed
ρ=0.39
Classical-like
ρ=-0.11
Neuro-like progenitor
ρ=0.36
Myc
ρ=-0.51
Latent time
Classical1
Classical2
Transitional
Classical/Basal hybrid
Basal/Hypoxia
EMT
Hypoxia
Neuroendocrine
Myc
Pancreatic myeloid
Duodenal tuft
G
Cumulative count
Kras barcode
(2,167,002/2,359,296
BC detected)
H
ρ=0.131
p-value=2.2x10^{-16}
Kras expression (scRNA-Seq)
Kras-hi (n=687)
Kras-lo (n=2,646)
Kras-neg (n=3,666)
Kras score (KrasBC-Seq)
ρ=0.439
p-value=2.2x10^{-16}
Kdm8 raw count (scRNA-Seq)
2 (n=15)
1 (n=450)
0 (n=6,534)
Kdm8 score (Kdm8-Seq)
I
Cell state
PTC 218
PTC 252
$Kras^{G12D}$ BC-defined clonotype
J
PTC308
PTC147
PTC218
PTC252
PTC254
Percent
100% 75% 50% 25% 0%
Severe
Medium
Mild
None
Imbalanced
Balanced
Somatic
Hypoxia level
K
Kdm8 state
Neg (n=6,202)
Pos (n=797)
Count
Normalized Kdm8 expression
L
Kras sig up
NES=1.75
FDR q=0.01
Enrichment score

Figure S4, related to Figure 4.

(A) Representative FACS density plots of the Tomato-positive, lineage (Cd31, Cd45, Ter119, and F4/80)-negative malignant cells from the primary PDA tumor, lung metastases, and ascites of a PTC^{Kdm8KO} mouse. The gate used to sort the Tomato+lineage- cells is shown. (B) Dot plot showing select differential genes across the identified Seurat cell clusters defined in Figure 4B. Dot size represents the percentage of cells expressing the differential genes and color represents the abundance of the differential genes. (C) Relative abundance of the previously reported malignant programs (Hwang et al, 2022 Nat Genet; Di Chiaro, et al, 2024 Cancer Cell; Moffitt et al, 2015 Nat Genet) and GSEA hallmark gene signatures across the 11 clusters of malignant cells defined in Figure 4B. (D) Dot plots demonstrating the Pearson correlation coefficient between any two malignant programs as in C quantified in the current study (left) and the bulk tumors from the PanCuRx PDA cohort (right). Select comparisons are highlighted with their Pearson correlation coefficients annotated. (E) Total cell counts on the indicated days following normoxic or hypoxic culture of 3 murine PDA cell lines expressing control or 2 *Kdm8*-targeting shRNAs. (F) Abundance of the indicated malignant programs plotted along the latent time and colored by the clusters defined in Figure 4B. Pearson correlation coefficients (ρ) are shown. (G) Cumulative plot for the AAV plasmid library. (H) Relationships between the transcript abundance measure by 10x scRNA-Seq (x-axis) and KrasBC-Seq (left, y-axis) or Kdm8-Seq (right, y-axis). Pearson correlation coefficients (ρ) and p-values are shown. (I) Sankey diagrams for all barcoded (bottom) malignant cells isolated from two PTC^{Kdm8KO} mice that correspond to the malignant programs defined in Figure 4B (top). (J) Proportions of malignant cells isolated from 5 PTC^{Kdm8KO} tumors, categorized by the indicated $Kras^{G12D}/Kras^{WT}$ allelic conformation, across varying levels of hypoxia (x-axis, from none to severe), quantified using the GSVA method. (K) Histogram of *Kdm8* expression in the malignant cells from 5 PTC^{Kdm8KO} tumors (n=6,999) estimated by Kdm8-Seq. (L) GSEA for the indicated hallmark gene signature (Kras signaling up) enriched in $Kdm8^{neg}$ malignant cells as in Figure 4L.

A

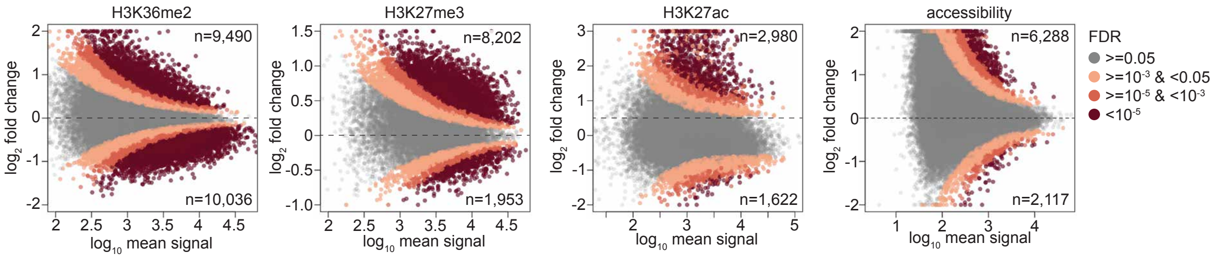

B

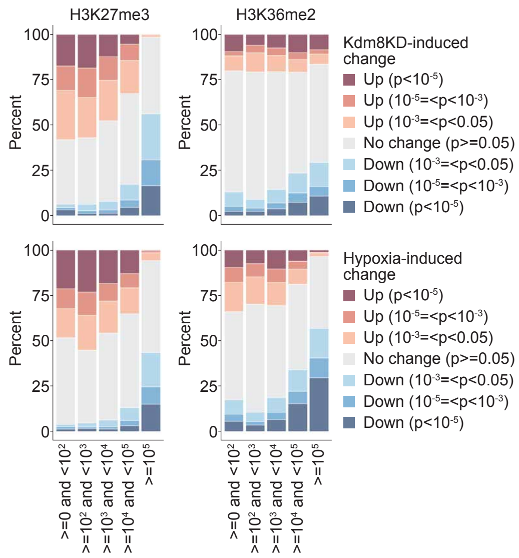

C

Dis to TSS	H3K36me2 Hypoxia-suppressed	H3K36me2 *Kdm8*KD-suppressed	H3K27me3 Hypoxia-suppressed	H3K27me3 *Kdm8*KD-suppressed
Proximal (<10^3 bp)	383/3,447 (11.1%)	320/3,447 (9.3%)	59/1,292 (4.6%)	82/1,292 (6.3%)
Distal (>=10^3 bp)	9,653/35,228 (27.4%)	6,820/35,228 (19.4%)	1,894/18,717 (10.1%)	2,468/18,717 (13.2%)
p-value	4.06×10^{-96}	7.91×10^{-48}	1.08×10^{-10}	1.37×10^{-12}
Dis to TSS	Hypoxia-induced	*Kdm8*KD-induced	Hypoxia-induced	*Kdm8*KD-induced
Proximal (<10^3 bp)	1,040/3,447 (30.2%)	712/3,447 (20.7%)	703/1,292 (54.4%)	738/1,292 (57.1%)
Distal (>=10^3 bp)	8,450/35,228 (24.0%)	7,240/35,228 (20.6%)	7,496/18,717 (40.0%)	7,439/18,717 (39.7%)
p-value	9.54×10^{-16}	0.9	4.34×10^{-24}	1.50×10^{-34}

D

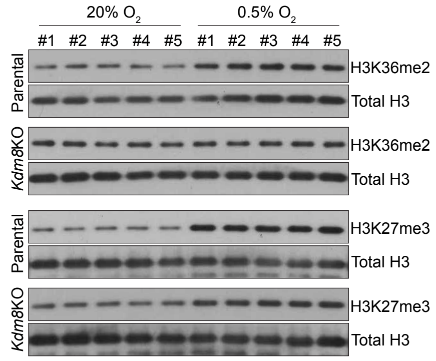

E

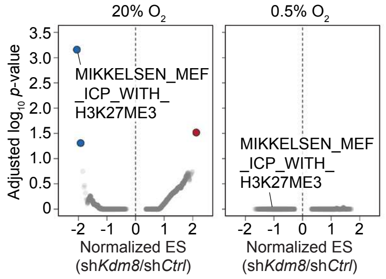

F

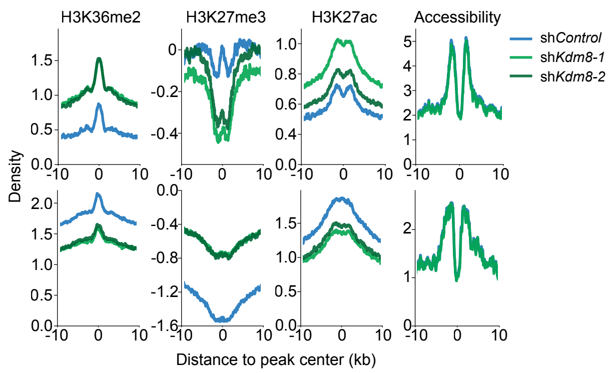

G

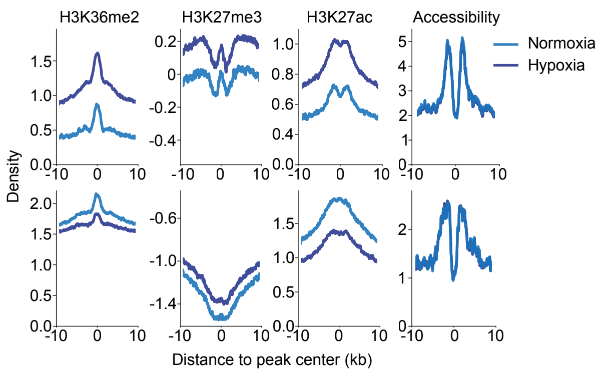

Figure S5, related to Figure 5.
(A) Hypoxia-induced differential H3K36me2, H3K27me3, and H3K27ac ChIP-Seq signals, as well as chromatin accessibility measured by ATAC-Seq (left to right), plotted against the mean read counts per region, as in Figure 5A. Dotted lines represent zero $\log_2$ fold change (FC), with positive values representing hypoxia-induced increases. FDR (false discovery rate) reflects the probability that a differential region exhibits a significant change induced by hypoxia. The number of significantly altered regions (FDR < 0.05) is indicated at the top and bottom of each plot. (B) Annotations of all H3K36me2 (right) and H3K27me3 (left) regions by Homer's annotatePeaks.pl, based on their distance to the nearest transcription start site (TSS), grouped into 5 bins with indicated genomic ranges in base pairs (x-axis). The y-axis shows the proportion of regions within each genomic range, stratified by the p-value of *Kdm8* knockdown (*Kdm8*KD)-induced (top) and hypoxia-induced (bottom) changes. (C) Fractions of proximal ($<10^3$ bp to TSS) and distal ($>=10^3$ bp to TSS) H3K36me2 (left) and H3K27me3 (right) genomic regions that are induced (bottom) or suppressed (top) by hypoxia or *Kdm8*KD. Chi-squared test p-values for the association between distance to TSS and *Kdm8*KD- or hypoxia-induced changes are shown. (D) Immunoblots showing H3K36me2, H3K27me3, and total H3 levels in parental 688M and a *Kdm8* knockout (*Kdm8*KO) clone cultured in normoxia or hypoxia (0.5% O_2). Data from 5 technical replicates per group are shown. (E) Gene set enrichment analysis (GSEA) of the curated gene sets (C2, v7.5.1) depleted in *Kdm8* knockdown 688M cells cultured in normoxia (left) or hypoxia (right). Normalized enrichment score (ES) for the gene signatures and the corresponding FDR-adjusted p-values are shown. sh*Ctrl*, control shRNA. The MEF cell derived gene signature bearing the H3K27me3 mark (Mikkelsen *et al*, 2008 Nature) is highlighted. (F,G) Aggregate signals of H3K36me2, H3K27me3, and H3K27ac ChIP-Seq, as well as chromatin accessibility measured by ATAC-Seq, within 3,973 genomic regions that are co-induced by *Kdm8*KD and hypoxia for H3K36me2 (top), and 3,838 regions that are co-suppressed by *Kdm8*KD and hypoxia for H3K36me2 (bottom), in control or sh*Kdm8*-expressing cells (F), and in 688M cells cultured in normoxia or hypoxia (G).

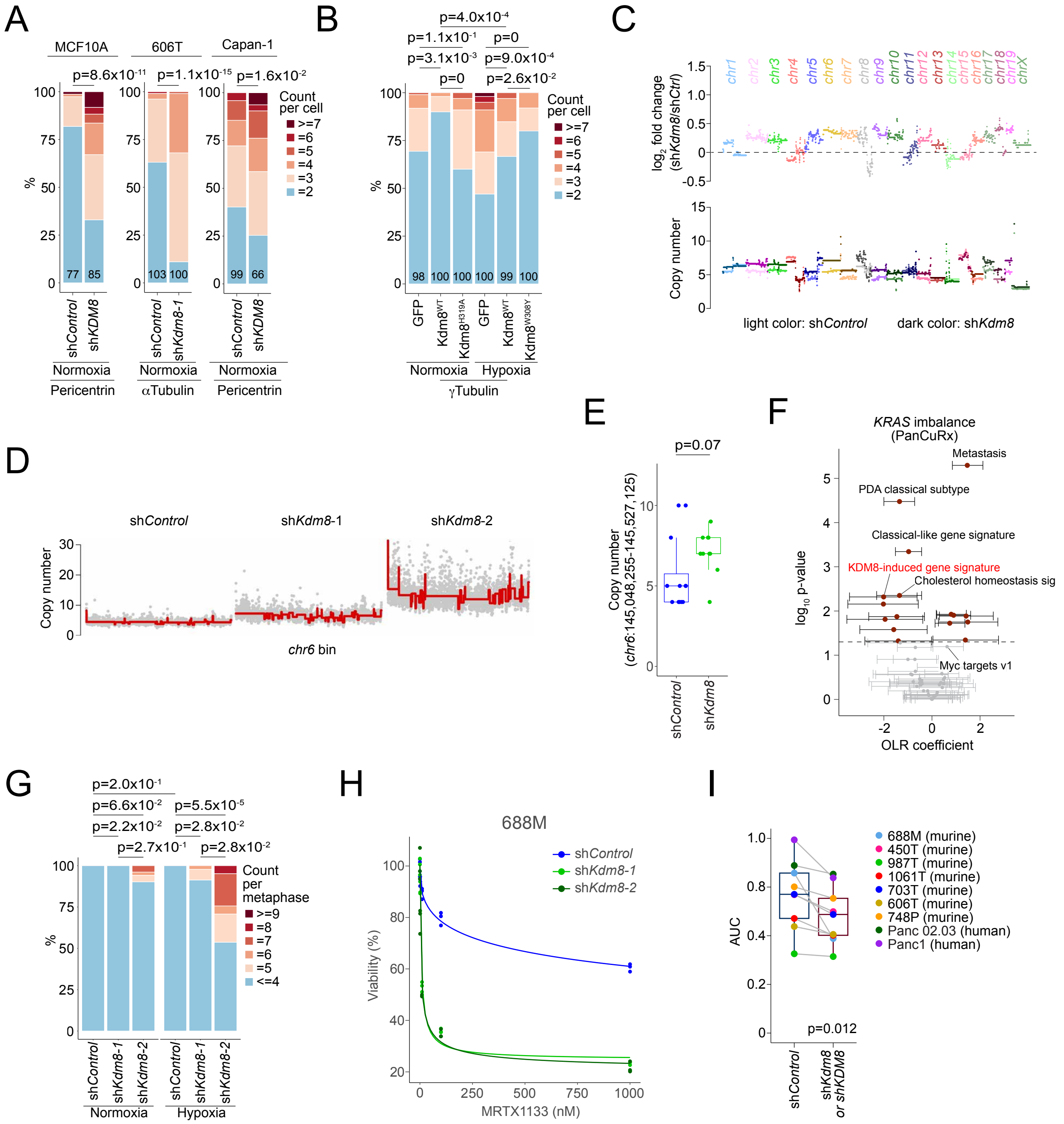

A
MCF10A
606T
Capan-1
p=8.6x10^-11
p=1.1x10^-15
p=1.6x10^-2
Count per cell
>=7
=6
=5
=4
=3
=2
%
77 85 103 100 99 66
shControl
shKDM8
shKdm8-1
Normoxia
Pericentrin
αTubulin
B
p=4.0x10^-4
p=1.1x10^-1
p=0
p=3.1x10^-3
p=9.0x10^-4
p=0
p=2.6x10^-2
98 100 100 100 99 100
GFP
Kdm8WT
Kdm8H319A
Kdm8W308Y
Normoxia
Hypoxia
γTubulin
C
log2 fold change (shKdm8/shCtrl)
Copy number
chr1 chr2 chr3 chr4 chr5 chr6 chr7 chr8 chr9 chr10 chr11 chr12 chr13 chr14 chr15 chr16 chr17 chr18 chr19 chrX
light color: shControl
dark color: shKdm8
D
shControl
shKdm8-1
shKdm8-2
Copy number
chr6 bin
E
p=0.07
Copy number (chr6:145,048,255-145,527,125)
shControl
shKdm8
F
KRAS imbalance (PanCuRx)
Metastasis
PDA classical subtype
Classical-like gene signature
KDM8-induced gene signature
Cholesterol homeostasis sig
Myc targets v1
log10 p-value
OLR coefficient
G
p=2.0x10^-1
p=6.6x10^-2
p=5.5x10^-5
p=2.2x10^-2
p=2.8x10^-2
p=2.7x10^-1
p=2.8x10^-2
Count per metaphase
>=9
=8
=7
=6
=5
<=4
shControl
shKdm8-1
shKdm8-2
Normoxia
Hypoxia
H
688M
shControl
shKdm8-1
shKdm8-2
Viability (%)
MRTX1133 (nM)
I
688M (murine)
450T (murine)
987T (murine)
1061T (murine)
703T (murine)
606T (murine)
748P (murine)
Panc 02.03 (human)
Panc1 (human)
AUC
p=0.012
shControl
shKdm8 or shKDM8

Figure S6, related to Figure 6.
(A) Quantifications of anaphase polarities by Pericentrin or αTubulin immunofluorescence (IF) in indicated control and *Kdm8*/*KDM8* deficient PDA cells as in Figure 6E. Numbers in the plot indicate the number of cells quantified in each group. (B) Proportions of *Kdm8* knockout 688M cells re-expressing indicated Kdm8 variants cultured in normoxia or hypoxia with indicated anaphase polarities quantified by γTubulin IF. Numbers in the plot indicate the number of cells quantified in each group. (C) Single-cell whole genome sequencing (scWGS) showing $\log_2$ fold change (sh*Kdm8*/sh*Control*, top) and the copy number of genomes in control (n=12) and sh*Kdm8*-expressing 688M cells (n=11, bottom). (D) Bulk whole genome sequencing showing chromosome 6 copy-number alterations in control and 688M cells expressing two sh*Kdm8*s. (E) Copy numbers of the indicated genomic location on chromosome 6 (mm39) where *Kras* is located in control and sh*Kdm8*-expressing 688M cells using scWGS as in (C). p-value = 0.07 using two-sided Wilcoxon test. (F) Ordinal logistic regression (OLR) for the association between the *KRAS* allelic imbalance (wt, 0; balanced, 1; minor, 2; major, 3) and the GSEA hallmark transcriptomic programs, anatomic location (primary site, 0; liver metastasis, 1), PDA subtype (classical, 1; basal, 0), and KDM8-induced gene signature in the PanCuRx PDA cohort. Coefficients with the 2.5/97.5% confidence intervals (x-axis) and $\log_{10}$ converted p-values (y-axis) are shown. KDM8-induced gene signature is highlighted. Dotted line represents statistical significance (p=0.05). (G) Quantifications of FISH as in Figure 6I using a chromosome 18 centromeric probe (yellow, Figure 6H) in control and sh*Kdm8*-expressing 688M cells cultured in normoxia and hypoxia. Proportions of control and the sh*Kdm8* metaphase 688M cells cultured in normoxia or hypoxia are shown. (H) Percent viability of 688M cells expressing control or *Kdm8*-targeting shRNAs treated with indicated concentrations of $Kras^{G12D}$ inhibitor MRTX1133. Results from three repeated experiments are shown. (I) Boxplot showing responses to MRTX1133 quantified by the area under the dose-response curve (AUC) for the indicated PDA cell lines. Significance was determined via paired student t-test following a Shapiro-Wilk test for normality.

Table S1. Survival and clinical features of disease progression in the PDA GEM model

Date of birth	Date of surgery	Date of death	Days	Surgical Procedure	Animal ID	Mouse genotype	Virus infused	Gender	Infused volume (uL)	PDA tumor	Splenomegaly	Ascites	Lung met #	Liver met #	Peritoneal met
3/17/22	5/6/22	2/16/23	286	Pancreatic ductal injection	147	PTC	AAV-U6-sgKdm8(J1)-KrasG12D(BC)	F	80	Y	Y	Y	26	0	Y
3/17/22	5/6/22	11/19/22	197	Pancreatic ductal injection	305	PTC	AAV-U6-sgKdm8(J1)-KrasG12D(BC)	F	110	N	NA	NA	NA	NA	NA
3/17/22	5/11/22	2/27/23	292	Pancreatic ductal injection	306	PTC	AAV-U6-sgKdm8(J1)-KrasG12D(BC)	M	10	N	NA	NA	NA	NA	NA
3/17/22	5/11/22	2/27/23	292	Pancreatic ductal injection	142	PTC	AAV-U6-sgKdm8(J1)-KrasG12D(BC)	M	100	N	NA	NA	NA	NA	NA
3/17/22	5/11/22	2/28/23	293	Pancreatic ductal injection	307	PTC	AAV-U6-sgKdm8(J1)-KrasG12D(BC)	F	70	Y	**Y**	Y	47	0	Y
3/24/22	5/11/22	11/4/22	177	Pancreatic ductal injection	308	PTC	AAV-U6-sgKdm8(J1)-KrasG12D(BC)	M	80	Y	**Y**	Y	8	NA	N
5/23/22	6/29/22	2/8/23	224	Pancreatic ductal injection	214	PTC	AAV-U6-sgKdm8(J1)-KrasG12D(BC)	M	100	Y	**Y**	Y	109	12	Y
5/23/22	6/29/22	10/27/22	120	Pancreatic ductal injection	215	PTC	AAV-U6-sgKdm8(J1)-KrasG12D(BC)	M	90	N	NA	NA	NA	NA	NA
5/23/22	6/29/22	12/9/22	163	Pancreatic ductal injection	216	PTC	AAV-U6-sgKdm8(J1)-KrasG12D(BC)	M	90	N	NA	NA	NA	NA	NA
5/23/22	6/29/22	1/2/23	187	Pancreatic ductal injection	217	PTC	AAV-U6-sgKdm8(J1)-KrasG12D(BC)	M	100	Y	N	Y	51	18	Y
5/23/22	6/29/22	1/27/23	212	Pancreatic ductal injection	218	PTC	AAV-U6-sgKdm8(J1)-KrasG12D(BC)	M	120	Y	**Y**	Y	39	4	Y
5/23/22	6/29/22	10/21/22	114	Pancreatic ductal injection	219	PTC	AAV-U6-sgKdm8(J1)-KrasG12D(BC)	M	130	N	NA	NA	NA	NA	NA
8/29/22	10/6/22	1/24/23	110	Pancreatic ductal injection	252	PTC	AAV-U6-sgKdm8(J1)-KrasG12D(BC)	F	50	Y	**Y**	Y	60	2	Y
8/29/22	10/6/22	2/18/23	135	Pancreatic ductal injection	253	PTC	AAV-U6-sgKdm8(J1)-KrasG12D(BC)	M	50	Y	**Y**	Y	25	1	Y
8/29/22	10/6/22	2/3/23	120	Pancreatic ductal injection	254	PTC	AAV-U6-sgKdm8(J1)-KrasG12D(BC)	M	50	Y	**Y**	N	20	0	Y
1/12/23	2/22/23	7/17/23	145	Pancreatic ductal injection	378	PTC	AAV-U6-sgKdm8(J1)-KrasG12D(BC)	M	100	N	NA	NA	NA	NA	NA
1/12/23	2/22/23	7/17/23	145	Pancreatic ductal injection	379	PTC	AAV-U6-sgKdm8(J1)-KrasG12D(BC)	M	50	N	NA	NA	NA	NA	NA
1/12/23	2/22/23	6/21/23	119	Pancreatic ductal injection	501	PTC	AAV-U6-sgKdm8(J1)-KrasG12D(BC)	M	50	Y	N	Y	32	2	Y
3/28/23	5/22/23	4/4/24	318	Pancreatic ductal injection	449	PTC	AAV-U6-sgNT-KrasG12D	M	40	Y	NA	N	6	0	N
3/28/23	5/22/23	12/5/23	197	Pancreatic ductal injection	450	PTC	AAV-U6-sgNT-KrasG12D	M	280	Y	NA	Y	8	0	N
4/3/23	5/22/23	11/17/23	179	Pancreatic ductal injection	488	PTC	AAV-U6-sgNT-KrasG12D	M	80	Y	Y	Y	15	0	Y
4/3/23	5/22/23	2/15/24	269	Pancreatic ductal injection	491	PTC	AAV-U6-sgNT-KrasG12D	M	30	Y	NA	N	18	0	N
4/22/23	5/22/23	12/26/23	218	Pancreatic ductal injection	606	PTC	AAV-U6-sgNT-KrasG12D	M	70	Y	Y	Y	7	0	N
4/22/23	5/22/23	1/2/24	225	Pancreatic ductal injection	608	PTC	AAV-U6-sgNT-KrasG12D	M	60	Y	Y	Y	24	0	N
4/22/23	5/22/23	11/28/23	190	Pancreatic ductal injection	609	PTC	AAV-U6-sgNT-KrasG12D	M	120	Y	Y	Y	12	0	N
4/22/23	5/22/23	4/4/24	318	Pancreatic ductal injection	612	PTC	AAV-U6-sgNT-KrasG12D	M	25	Y	Y	Y	88	0	N
4/22/23	5/22/23	11/7/23	169	Pancreatic ductal injection	613	PTC	AAV-U6-sgNT-KrasG12D	M	40	Y	NA	NA	NA	NA	NA
10/24/24	12/10/24	4/23/25	134	Pancreatic ductal injection	2445	PTC	AAV-U6-sgNT-KrasG12D	F	20	Y	Y	Y	18	0	Y
10/24/24	12/10/24	4/25/25	136	Pancreatic ductal injection	2446	PTC	AAV-U6-sgNT-KrasG12D	F	50	Y	Y	Y	22	0	N
10/30/24	12/10/24	4/23/25	134	Pancreatic ductal injection	2429	PTC	AAV-U6-sgNT-KrasG12D	F	50	Y	Y	Y	1	0	N
10/31/24	12/10/24	4/7/25	118	Pancreatic ductal injection	2487	PTC	AAV-U6-sgNT-KrasG12D	F	70	Y	Y	N	77	0	N
